# Incidence and risk factors for suicide and attempted suicide following a diagnosis of hematological malignancy

**DOI:** 10.1002/cam4.316

**Published:** 2014-08-26

**Authors:** Malin Hultcrantz, Tobias Svensson, Åsa R Derolf, Sigurdur Y Kristinsson, Ebba K Lindqvist, Anders Ekbom, Fredrik Granath, Magnus Björkholm

**Affiliations:** 1 Division of Hematology, Department of Medicine, Karolinska University Hospital Solna and Karolinska InstitutetStockholm, Sweden; 2 Clinical Epidemiology Unit, Department of Medicine, Karolinska University Hospital Solna and Karolinska InstitutetStockholm, Sweden; 3 Faculty of Medicine, University of Iceland and Department of Hematology, Landspitali National University HospitalReykjavik, Iceland

**Keywords:** Multiple myeloma, population based, psychiatric disorder, suicide, suicide attempt

## Abstract

Solid tumors are associated with an increased risk of suicide, however, there is limited detailed information on the risk of suicide in patients with hematological malignancies. Therefore, we conducted a population-based study including 47,220 patients with hematological malignancies (diagnosed 1992–2006) and their 235,868 matched controls to define the incidence and risk factors for suicide and suicide attempt. Information on suicides, suicide attempts, and preexisting psychiatric disorders was obtained from Swedish registers and individual medical records. There was a twofold increased (hazard ratio [HR] = 1.9, 95% confidence interval 1.5–2.3, *P* < 0.0001) risk of suicide/suicide attempt during the first 3 years after diagnosis in patients with hematological malignancies compared to matched controls. Of all hematological malignancies, multiple myeloma was associated with the highest risk (HR = 3.4; 2.3–5.0, *P* < 0.0001). Patients with a preexisting psychiatric disorder were at a very high risk of suicide and suicide attempt (HR = 23.3; 16.6–32.6, *P* < 0.0001), regardless of type of hematological malignancy. Among patients who committed suicide, 19% were in a palliative phase and 44% were in remission with no active treatment. In conclusion, the risk of suicide and suicide attempt is elevated in patients with hematological malignancies. Certain high-risk patients may benefit from early detection and preventive measures.

## Introduction

Survival and cure rates have increased in most hematological malignancies, mainly due to improved disease-specific treatment options and better supportive care 1–6. Many hematological malignancies are, however, still associated with a dismal prognosis. In addition, the diseases themselves as well as their treatment can affect the mental health and quality of life of these patients 7–10. An elevated risk of suicides has been observed in cancer patients compared to the general population 11,12. In patients with solid cancers, risk factors such as male gender, respiratory, and upper GI-tract cancers, as well as advanced disease stage, have been associated with a high suicide risk 11–14. A few studies where patients with hematological malignancies have been included in large subgroups have reported an increased risk of suicide 11,13. However, there is limited detailed information on the risk and risk factors for suicide and suicide attempt in patients with different hematological malignancies. Therefore, we conducted a large population-based study to assess the incidence and risk factors for suicide and suicide attempt in patients with a broad range of hematological malignancies.

## Materials and Methods

### Central registers, patients, and controls

Sweden, a country of 9.5 million people, provides universal health care for its entire population. The Swedish Cancer Register was established in 1958 15 and has a high level of coverage for hematological malignancies 16. It is mandatory for every physician and pathologist/cytologist to report all incident cases of malignant disease to the Cancer Register. In Sweden, all dates and causes of death are reported to the Cause of Death Register. Since 1964, the centralized Swedish Inpatient Register holds information on all somatic and psychiatric hospital discharge diagnoses with a high level of coverage 17.

The Swedish Cancer Register was used to identify all individuals diagnosed with a hematological malignancy between 1st January 1992 and 31st December 2006. Patients under the age of 18 years at diagnosis and patients with any other malignancy prior to the hematological malignancy were excluded. For each patient, five population-based controls, matched by gender, year of birth, and county of residence, were chosen randomly from the Swedish Register of Total Population. All controls had to be alive and free of hematologic or nonhematologic malignancy at the time of diagnosis for the corresponding patient. Controls were censored at time of death of the corresponding patient.

Information on suicides was obtained from the Cause of Death Register and hospital admissions due to suicide attempts were identified through the Inpatient Register. Suicide and suicide attempt were defined as *Intentional self-harm X60-X84* from ICD10 and *Suicide and self-inflicted injury E950-E959* from ICD9. *Events of undetermined intent Y10-Y34* (ICD10) and *Injury Undetermined whether accidental or purposely inflicted E980-E989* (ICD9) were also included since a substantial portion of these deaths are considered to be suicides 18,19. Information on admissions due to psychiatric disorders, defined as at least one admission with any psychiatric diagnosis prior to the cancer diagnosis, was obtained from the Inpatient Register.

For patients who committed suicide during the first 3 years after diagnosis, detailed information on patient characteristics, disease type and stage, as well as treatment was collected from the patient medical records. The presence of pain was defined as pain complaints being noted in the medical record and/or treatment with continuous analgesics.

The study was approved by the Stockholm Regional Ethics Review Board. Informed consent was waived, because we had no contact with study patients and the data used for analyses did not contain any personal identifiers.

### Statistical analysis

Patients and controls were followed from the date of diagnosis or the corresponding time for the controls, until death, emigration, or end of follow-up. Suicide attempts were assessed until 31st December 2006 and suicides until 31st December 2005 due to delayed reporting to the Cause of Death Register. Cox regression was used to analyze the risk of suicide and suicide attempt and results are presented at hazard ratios (HRs) with 95% confidence intervals (CIs). The HRs for suicide and suicide attempt were analyzed in relation to follow-up time after diagnosis, age, and gender. Separate analyses were performed for the different types of hematological malignancies: non-Hodgkin lymphoma (NHL), Hodgkin lymphoma (HL), multiple myeloma (MM), acute leukemia, including acute myeloid leukemia (AML) and acute lymphoblastic leukemia (ALL), and chronic lympho- and myeloproliferative disorders, including chronic lymphocytic leukemia (CLL), chronic myeloid leukemia (CML), and myeloproliferative neoplasms (MPNs).

The HRs for suicide and suicide attempt were nearly identical in all separate and combined analysis and are therefore presented as a combined end point if not otherwise specified. In addition, incidence of suicide and suicide attempt per thousand person-years of follow-up were calculated in relation to preexisting psychiatric disorders in both patients and controls.

## Results

A total of 47,220 cases and 235,868 controls were identified between 1st January 1992 and 31st December 2006. Of these, 54.6% were men and the median age at diagnosis was 70 years (range 18–102 years; Table1). Median follow-up was 32 months. The number of patients alive at 3 years after diagnosis was 28,459 and 18,258 patients were alive at the end of follow-up.

**Table 1:** Characteristic of patients with a hematological malignancy and their matched controls.

	Patients			Controls		
	*N *=* *47,220	%	Number of suicides/suicide attempts	*N *=* *235 868	%	Number of suicides/suicide attempts
Gender						
Women	21,453	45.4	96	107,059	45.4	461
Men	25,767	54.6	116	128,809	54.6	651
Age at diagnosis						
18–29	1394	3.0	21	6587	2.8	58
30–39	1602	3.4	13	8008	3.4	64
40–49	3056	6.5	24	15,278	6.5	115
50–59	6628	14.0	38	33,136	14.0	173
60–69	10,384	22.0	39	51,911	22.0	203
70–79	14,786	31.3	49	73,910	31.3	324
80–89	8619	18.3	27	43,073	18.3	168
90 and older	751	1.6	1	3965	1.7	7
Calendar year of diagnosis						
1992–1994	8652	18.3	54	43,223	18.3	355
1995–1997	9153	19.4	62	45,688	19.4	314
1998–2000	9440	20.0	42	47,198	20.0	220
2001–2003	9963	21.1	37	49,714	21.1	173
2004–2006	10,012	21.2	17	50,045	21.2	50
Type of hematological malignancy						
Non-Hodgkin lymphoma	18,583	39.3	46	NA	NA	NA
Hodgkin lymphoma	2248	4.8	11	NA	NA	NA
Multiple myeloma	7778	16.5	37	NA	NA	NA
Acute leukemia (AML/ALL)	3746	7.9	9	NA	NA	NA
CLL/CML/MPN	14,865	31.5	33	NA	NA	NA
Preexisting psychiatric disorder^1^						
Yes	2042	4.3	57	11,027	4.7	262
No	45,178	95.7	155	224,841	95.3	850

AML, acute myeloid leukemia;

ALL, acute lymphoblastic leukemia;

CLL, chronic lymphocytic leukemia;

CML, chronic myeloid leukemia;

MPN, myeloproliferative neoplasm;

NA, not applicable.

1 Preexisting psychiatric disorder was defined as a hospital admission with a psychiatric diagnosis prior to the diagnosis of the hematological malignancy.

In total, there were 54 suicides and 158 suicide attempts among patients (Table2), of which 36 and 100 occurred during the first 3 years after diagnosis, respectively. The risk of suicide and suicide attempt was twice as high in patients with hematological malignancies compared to matched controls during the first 3 years after diagnosis (HR = 1.9, 95% CI 1.5–2.3, *P* < 0.0001). The excess risk was modified by time after diagnosis and no remaining risk-increase was observed when more than 3 years had elapsed (HR = 1.1, 0.9–1.4, *P* = 0.3). In separate analysis including only consummated suicides, identical risks were observed (HR = 1.9, 1.3–2.8 *P* = 0.0005) during the first 3 years after diagnosis, and HR 1.2 (0.8–1.8, *P* = 0.4) after four or more years of follow-up (Table2). Due to the very similar HRs for suicide, suicide attempt, and the combined end point suicide and suicide attempt, the following results represent the combined end point suicide/suicide attempt occurring during the first 3 years after diagnosis unless otherwise specified.

**Table 2:** Suicides and suicides attempt in relation to time after diagnosis in patients compared to matched controls.

	Number of events among patients	HR	95% CI	*P*-value
Suicides/suicide attempts	212	1.5	1.3–1.8	<0.0001
Suicides	54	1.6	1.2–2.1	0.0018
Suicides/suicide attempts ≤3 years after diagnosis	136	1.9	1.5–2.3	<0.0001
Suicides ≤3 years after diagnosis	36	1.9	1.3–2.8	0.0005
Suicides/suicide attempts >3 years after diagnosis	76	1.1	0.9–1.5	0.29
Suicides >3 years after diagnosis	18	1.2	0.8–1.8	0.38

HR, hazard ratio;

CI, confidence interval.

MM was associated with the highest risk of suicide and suicide attempt (HR = 3.4; 2.3–5.0 *P* < 0.0001) during the first 3 years after diagnosis. The risk was also significantly elevated for patients with NHL (HR = 1.7; 1.2–2.3 *P* = 0.002). In patients with HL, acute leukemia, and CLL/CML/MPN there was a tendency toward an increased risk, however, this was not statistically significant (Table3).

**Table 3:** Risk of suicide/suicide attempt in relation to calendar period, gender, age, type of hematological malignancy, and history of psychiatric disorder during the first 3 years after diagnosis.

	Year 0–3 of follow-up after diagnosis			
	Number of events among patients	HR	95% CI	*P*-value
Gender				
Male	72	1.7	1.3–2.2	
Female	64	2.1	1.5–2.7	0.38
Age at diagnosis				
≤69	63	1.9	1.5–2.5	
≥70	73	1.8	1.3–2.4	0.76
Calendar period of diagnosis				
1992–1998	80	2.2	1.7–2.9	
1999–2006	56	1.6	1.2–2.1	0.065
Type of hematological malignancy				
Non-Hodgkin lymphoma	46	1.7	1.2–2.3	
Hodgkin lymphoma	11	1.8	0.9–3.6	
Multiple myeloma	37	3.4	2.3–5.0	
Acute leukemia (AML/ALL)	9	1.9	0.9–4.1	
CLL/CML/MPN	33	1.5	0.99–2.1	0.001^1^

HR, hazard ratio;

CI, confidence interval;

AML, acute myeloid leukemia;

ALL, acute lymphoblastic leukemia;

CLL, chronic lymphocytic leukemia;

CML, chronic myeloid leukemia;

MPN, myeloproliferative neoplasm.

1 Test of homogeneity. This significance is mainly attributed to a higher risk for multiple myeloma.

**Figure 1:**
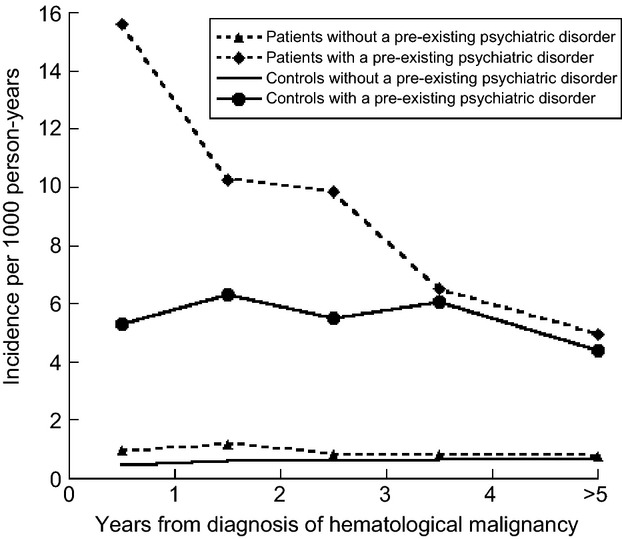
Suicide and suicide attempt among patients and controls with and without a preexisting psychiatric disorder per year, starting at diagnosis shown as incidence/1000 person-years.

There was a multiplicative interaction regarding the risk of suicide/suicide attempt in patients with a preexisting psychiatric disorder and a hematological malignancy. Compared to controls without a preexisting psychiatric disorder, the HRs of suicide/suicide attempt in patients with a preexisting psychiatric disorder was 23.3 (16.6–32.6, *P* < 0.0001), patients without preexisting psychiatric disorder HR = 1.8 (1.4–2.3, *P* < 0.0001) and controls with a preexisting psychiatric disorder HR = 10.8 (8.8–13.2, *P* < 0.0001) during the first 3 years of follow-up (Table4). A similar pattern was observed when the incidence of suicides/suicide attempts per 1000 person-years was calculated in patients and controls with and without a preexisting psychiatric disorder (Fig.1).

**Table 4:** Risk of suicide/suicide attempt in relation to history of psychiatric disorder during the first 3 years after diagnosis of the hematological malignancy.

	Year 0–3 of follow-up after diagnosis			
	Number of events among patients	HR	95% CI	*P*-value
Patients without a preexisting psychiatric disorder (*n* = 45,178)	92	1.8	1.4–2.3	<0.0001
Patients with a preexisting psychiatric disorder (*n* = 2042)	44	23.3	16.7–32.6	<0.0001
Controls without a preexisting psychiatric disorder (*n* = 224,841)	325	1.0	NA	Reference
Controls with a preexisting psychiatric disorder (*n* = 11,027)	147	10.8	8.3–13.2	<0.0001

HR, hazard ratio;

CI, confidence interval;

NA, not applicable.

The excess risk of suicide/suicide attempt associated with hematological malignancy was not significantly modified by age at diagnosis. The HR for patients ≤69 years at diagnosis was 1.9 (1.5–2.5) and for patients ≥70 years at diagnosis was 1.8 (1.3–2.4; Table3). The absolute number of suicides/suicide attempts was higher in males, however, the relative risks were similar in the two genders. The HRs were 1.7 (1.3–2.2) and 2.1 (1.5–2.7; *P* = 0.38 for difference), in males and females, respectively (Table3). There was a trend toward a lower risk in patients diagnosed between 1999 and 2006 (HR = 1.6; 1.2–2.1) compared to patients diagnosed between 1992 and 1998 (HR = 2.2; 1.7–2.9) although this difference was not statistically significant (*P* = 0.07; Table3).

Thirty-six patients committed suicide during the first 3 years after diagnosis, of which 23 had NHL, nine had MM, two had AML, and two had polycythemia vera. Twelve patients (33%) were on active treatment which included corticosteroids in ten patients (28%). Sixteen patients (44%) were in remission with no ongoing treatment and seven patients (19%) were in a palliative phase. Pain was noted in the medical record of 14 patients (39%). Seventeen patients (47%) had a preexisting psychiatric disorder and nine patients (25%) had attempted to commit suicide prior to their cancer diagnosis (Table5). The most commonly used methods of suicide were hanging and intoxication. Suicides including firearms were only seen in men.

**Table 5:** Demography, disease characteristics, and suicide methods in patients who committed suicide during the first 3 years after diagnosis

Patients	Number	
Median age at diagnosis	68.5 (years)	
Median age at suicide	69 (years)	
Men	23	
Women	13	
Type of hematological malignancy		
Multiple myeloma	9	
Non-Hodgkin lymphoma	23	
Acute myeloid leukemia	2	
Polycythemia vera	2	
Patient and disease characteristics	Yes	No
Preexisting psychiatric disorder	17	18
Previous suicide attempt	9	26
Active treatment	12	23
Including corticosteroids	10	25
In remission with no ongoing treatment	16	19
Palliation	7	28
Substantial pain	14	21
Unknown *n* = 1		
Method of suicide	Men	Women
Hanging	6	4
Intoxication with sedatives/analgesics	5	3
Fall/jump	4	2
Drowning	1	4
Firearm	4	–
Burn/fire	3	–
Total	23	13

## Discussion

In this large population-based study we found the risk of suicide and suicide attempt to be twice as high in patients with hematological malignancies compared to matched controls. The risk was elevated during the first 3 years after diagnosis while no excess risk was observed when more than 3 years had elapsed. The highest risks were observed in patients with MM and in patients with a preexisting psychiatric disorder irrespective of type of hematological malignancy. Even though the absolute number of suicides/suicide attempts was limited, there are patients at high risk of committing suicide who may benefit from early detection and preventive measures.

Patients with a hematological malignancy were at a higher risk of committing suicide/suicide attempt shortly after diagnosis. In a recent Swedish study, the risk of suicide and cardiovascular death was significantly elevated during the first year, even the first weeks, after receiving a diagnosis of cancer emphasizing that a cancer diagnosis is a traumatic experience associated with both emotional and physical stress 20. Similar temporal patterns have been observed in large studies on cancer patients where patients with hematological malignancies have been included 11–13. After more than 3 years following diagnosis, patients who are still alive may either be cured or may have adapted emotionally to having a malignant disease. The awareness of suicidal ideation in patients should be higher among health care personnel shortly after the cancer diagnosis.

MM was the hematological malignancy associated with the highest risk of suicide/suicide attempt. MM is in the majority of cases an incurable disease and often associated with debilitating bone lesions and pain 8,21. Also, the treatment can have a negative impact on quality of life; high doses of corticosteroids, included in most MM treatment regimen, can induce depression and psychosis and thus enhance the risk of suicide/suicide attempt 22,23. There are recent studies indicating that early treatment, before organ damage is evident, may improve survival in MM patients 24. Optimizing MM management, possibly by more effective novel therapies earlier in the disease course, and better psychological care is of great importance for improving survival and quality of life in MM patients.

An elevated risk of suicide/suicide attempt was also observed in patients with NHL while HL, acute leukemias and CLL/CML/MPN were all associated with a nonsignificant increase in risk of suicide/suicide attempt. There was also a trend toward a lower risk of suicide/suicide attempt toward the second calendar period. In previous studies, patients with aggressive diseases and poor prognosis such as acute leukemias have been observed to have an elevated risk of suicide/suicide attempt 12,25. New treatment options have emerged for many of the hematological malignancies during the current study period 4,5,26. The majority of the recently introduced treatments have a favorable effect on both survival and quality of life but their potential impact on risk of suicide and suicidal ideation needs to be elucidated.

Patients with a preexisting psychiatric disorder had a substantially elevated risk of suicide/suicide attempt compared to controls regardless of type of hematological malignancy. Psychiatric disorders including depression should be recognized as a major risk factor for suicide/suicide attempt in patients with hematological malignancies. In addition, depression is more common in cancer patients and has been shown to correlate with more rapid cancer progression 27–31. Interestingly, there are hypotheses of an immunological interaction between cancer and depression. Dysregulation of the hypothalamo–pituitary–adrenal axis and production of proinflammatory cytokines have been shown to correlate both with depression and cancer 32–34. Especially interleukin-6 has been correlated to MM progression as well as to cancer-related depression and fatigue 35,36. This potential immunological relationship needs to be further clarified and if the association is shown to be causative, it may have great implications for the treatment of various cancers and cancer-related depression.

The first step of suicide prevention is to identify patients at an increased risk, which in our study were patients who were recently diagnosed, patients with MM, and patients with a preexisting psychiatric disorder. In the detailed analysis of the 36 patients who committed suicide, preexisting psychiatric disorders and previous suicide attempts were common. Depression was the most common psychiatric disorder, followed by alcohol abuse. Additional risk factors observed in previous studies are male gender, poor social support, emotional distress, pain, and advanced disease stage 27,33,37. The 14 of the 36 patients who were in remission with no preexisting psychiatric disorder would have been harder to identify at an earlier stage. Therefore, a high awareness of suicidal ideation in patients shortly after diagnosis but also during follow-up is important. Early identification of high-risk patients is valuable since effective treatment in multidisciplinary teams, including psychological intervention and antidepressive treatment, can lead to a better quality of life and may reduce the risk of suicide and suicide attempt 29,31,37,38.

Strengths of this study include the population-based design and a large number of patients during a period of 16 years. In addition, the study includes information from both high-quality Swedish registers as well as detailed information from individual medical records. One limitation is the possibility of underreporting of suicides in cancer patients. Suicides in cancer patients may go unnoticed and in addition, doctors may be reluctant to report suicides in terminally ill patients. The number of suicide attempts and the prevalence of preexisting psychiatric disorders may be underestimated since the Inpatient Register does not include patients treated in the outpatient setting. Thus, less severe suicide attempts and milder psychiatric disorders were not included and the study was instead focused on patients/controls with significant suicide attempts and more pronounced psychiatric disorders. Moreover, psychiatric complaints emerging after the cancer diagnosis are not analyzed in this study.

In summary, in this large population-based study we found patients with hematological malignancies, especially MM, to have a higher risk of suicide and suicide attempt compared to matched controls during the first 3 years following diagnosis. The risk elevation was strongly associated with a preexisting psychiatric disorder. Future studies are required to elucidate underlying neuropsychiatric and immunological causes, which may be shared in depression and cancer. Even though suicides/suicide attempts were rare events, awareness of risk factors such as short time since diagnosis, MM diagnosis, male gender, advanced disease stage, and a preexisting psychiatric disorder is important. Our results emphasize, in addition to optimal treatment of the malignant disease, the need for psychological assessment and early detection of cancer patients at high risk of committing suicide in order to enable effective prevention of suicides/suicide attempts.

## References

[b1] Derolf AR, Kristinsson SY, Andersson TM, Landgren O, Dickman PW, Bjorkholm M (2009). Improved patient survival for acute myeloid leukemia: a population-based study of 9729 patients diagnosed in Sweden between 1973 and 2005. Blood.

[b2] Kristinsson SY, Landgren O, Dickman PW, Derolf AR, Bjorkholm M (2007). Patterns of survival in multiple myeloma: a population-based study of patients diagnosed in Sweden from 1973 to 2003. J. Clin. Oncol.

[b3] Hultcrantz M, Kristinsson SY, Andersson TM, Landgren O, Eloranta S, Derolf AR (2012). Patterns of survival among patients with myeloproliferative neoplasms diagnosed in Sweden from 1973 to 2008: a population-based study. J. Clin. Oncol.

[b4] Coiffier B, Lepage E, Briere J, Herbrecht R, Tilly H, Bouabdallah R (2002). CHOP chemotherapy plus rituximab compared with CHOP alone in elderly patients with diffuse large-B-cell lymphoma. N. Engl. J. Med.

[b5] Bjorkholm M, Derolf AR, Hultcrantz M, Kristinsson SY, Ekstrand C, Goldin LR (2011). Treatment-related risk factors for transformation to acute myeloid leukemia and myelodysplastic syndromes in myeloproliferative neoplasms. J. Clin. Oncol.

[b6] Kristinsson SY, Anderson WF, Landgren O (2014). Improved long-term survival in multiple myeloma up to the age of 80 years. Leukemia.

[b7] Mohamedali H, Breunis H, Timilshina N, Brandwein JM, Gupta V, Minden MD (2012). Older age is associated with similar quality of life and physical function compared to younger age during intensive chemotherapy for acute myeloid leukemia. Leuk. Res.

[b8] Mols F, Oerlemans S, Vos AH, Koster A, Verelst S, Sonneveld P (2012). Health-related quality of life and disease-specific complaints among multiple myeloma patients up to 10 yr after diagnosis: results from a population-based study using the PROFILES registry. Eur. J. Haematol.

[b9] Mesa RA, Niblack J, Wadleigh M, Verstovsek S, Camoriano J, Barnes S (2007). The burden of fatigue and quality of life in myeloproliferative disorders (MPDs): an international Internet-based survey of 1179 MPD patients. Cancer.

[b10] Phillips KM, Pinilla-Ibarz J, Sotomayor E, Lee MR, Jim HS, Small BJ (2013). Quality of life outcomes in patients with chronic myeloid leukemia treated with tyrosine kinase inhibitors: a controlled comparison. Support. Care Cancer.

[b11] Misono S, Weiss NS, Fann JR, Redman M, Yueh B (2008). Incidence of suicide in persons with cancer. J. Clin. Oncol.

[b12] Bjorkenstam C, Edberg A, Ayoubi S, Rosen M (2005). Are cancer patients at higher suicide risk than the general population?. Scand. J. Public Health.

[b13] Hem E, Loge JH, Haldorsen T, Ekeberg O (2004). Suicide risk in cancer patients from 1960 to 1999. J. Clin. Oncol.

[b14] Innos K, Rahu K, Rahu M, Baburin A (2003). Suicides among cancer patients in Estonia: a population-based study. Eur. J. Cancer.

[b15] Sverige. Socialstyrelsen (2012). Cancer incidence in Sweden 2011 = Cancerförekomst i Sverige 2011.

[b16] Turesson I, Linet MS, Bjorkholm M, Kristinsson SY, Goldin LR, Caporaso NE (2007). Ascertainment and diagnostic accuracy for hematopoietic lymphoproliferative malignancies in Sweden 1964–2003. Int. J. Cancer.

[b17] Ludvigsson JF, Andersson E, Ekbom A, Feychting M, Kim JL, Reuterwall C (2011). External review and validation of the Swedish national inpatient register. BMC Public Health.

[b18] Allebeck P, Bolund C (1991). Suicides and suicide attempts in cancer patients. Psychol. Med.

[b19] Tanaka H, Tsukuma H, Masaoka T, Ajiki W, Koyama Y, Kinoshita N (1999). Suicide risk among cancer patients: experience at one medical center in Japan, 1978–1994. Jpn. J. Cancer Res.

[b20] Fang F, Fall K, Mittleman MA, Sparen P, Ye W, Adami HO (2012). Suicide and cardiovascular death after a cancer diagnosis. N. Engl. J. Med.

[b21] Kendal WS (2007). Suicide and cancer: a gender-comparative study. Ann. Oncol.

[b22] Eshaghian S, Berenson JR (2012). Multiple myeloma: improved outcomes with new therapeutic approaches. Curr. Opin. Support. Palliat. Care.

[b23] Fardet L, Petersen I, Nazareth I (2012). Suicidal behavior and severe neuropsychiatric disorders following glucocorticoid therapy in primary care. Am. J. Psychiatry.

[b24] Mateos MV, Hernandez MT, Giraldo P, de la Rubia J, de Arriba F, Lopez Corral L (2013). Lenalidomide plus dexamethasone for high-risk smoldering multiple myeloma. N. Engl. J. Med.

[b25] Miccinesi G, Crocetti E, Benvenuti A, Paci E (2004). Suicide mortality is decreasing among cancer patients in Central Italy. Eur. J. Cancer.

[b26] Richardson PG, Sonneveld P, Schuster MW, Irwin D, Stadtmauer EA, Facon T (2005). Bortezomib or high-dose dexamethasone for relapsed multiple myeloma. N. Engl. J. Med.

[b27] Breitbart W, Rosenfeld B, Pessin H, Kaim M, Funesti-Esch J, Galietta M (2000). Depression, hopelessness, and desire for hastened death in terminally ill patients with cancer. JAMA.

[b28] Henriksson MM, Isometsa ET, Hietanen PS, Aro HM, Lonnqvist JK (1995). Mental disorders in cancer suicides. J. Affect. Disord.

[b29] Tiernan E, Casey P, O'Boyle C, Birkbeck G, Mangan M, O'Siorain L (2002). Relations between desire for early death, depressive symptoms and antidepressant prescribing in terminally ill patients with cancer. J. R. Soc. Med.

[b30] Bolund C (1985). Suicide and cancer: II. Medical and care factors in suicides by cancer patients in Sweden, 1973–1976. J. Psychosoc. Oncol.

[b31] Spiegel D, Giese-Davis J (2003). Depression and cancer: mechanisms and disease progression. Biol. Psychiatry.

[b32] Schiepers OJ, Wichers MC, Maes M (2005). Cytokines and major depression. Prog. Neuropsychopharmacol. Biol. Psychiatry.

[b33] Spoletini I, Gianni W, Caltagirone C, Madaio R, Repetto L, Spalletta G (2011). Suicide and cancer: where do we go from here?. Crit. Rev. Oncol. Hematol.

[b34] O'Donovan A, Rush G, Hoatam G, Hughes BM, McCrohan A, Kelleher C (2013). Suicidal ideation is associated with elevated inflammation in patients with major depressive disorder. Depress. Anxiety.

[b35] Klein B, Zhang XG, Lu ZY, Bataille R (1995). Interleukin-6 in human multiple myeloma. Blood.

[b36] Boland E, Eiser C, Ezaydi Y, Greenfield DM, Ahmedzai SH, Snowden JA (2013). Living with advanced but stable multiple myeloma: a study of the symptom burden and cumulative effects of disease and intensive (hematopoietic stem cell transplant-based) treatment on health-related quality of life. J. Pain Symptom Manage.

[b37] Filiberti A, Ripamonti C (2002). Suicide and suicidal thoughts in cancer patients. Tumori.

[b38] Rehse B, Pukrop R (2003). Effects of psychosocial interventions on quality of life in adult cancer patients: meta analysis of 37 published controlled outcome studies. Patient Educ. Couns.

